# Return of spontaneous Circulation Is Not Affected by Different Chest Compression Rates Superimposed with Sustained Inflations during Cardiopulmonary Resuscitation in Newborn Piglets

**DOI:** 10.1371/journal.pone.0157249

**Published:** 2016-06-15

**Authors:** Elliott S. Li, Po-Yin Cheung, Tze-Fun Lee, Min Lu, Megan O'Reilly, Georg M. Schmölzer

**Affiliations:** 1 Faculty of Science, McGill University, Montreal, Quebec, Canada; 2 Centre for the Studies of Asphyxia and Resuscitation, Neonatal Research Unit, Royal Alexandra Hospital, Edmonton, AB, Canada; 3 Department of Pediatrics, University of Alberta, Edmonton, Alberta, Canada; University Hospital Medical Centre, GERMANY

## Abstract

**Objective:**

Recently, sustained inflations (SI) during chest compression (CC) have been suggested as an alternative to the current approach during neonatal resuscitation. However, the optimal rate of CC during SI has not yet been established. Our aim was to determine whether different CC rates during SI reduce time to return of spontaneous circulation (ROSC) and improve hemodynamic recovery in newborn piglets with asphyxia-induced bradycardia.

**Intervention and measurements:**

Term newborn piglets were anesthetized, intubated, instrumented and exposed to 45-min normocapnic hypoxia followed by asphyxia. Resuscitation was initiated when heart rate decreased to 25% of baseline. Piglets were randomized into three groups: CC superimposed by SI at a rate of 90 CC per minute (SI+CC 90, n = 8), CC superimposed by SI at a rate of 120 CC per minute (SI+CC 120, n = 8), or a sham group (n = 6). Cardiac function, carotid blood flow, cerebral oxygenation and respiratory parameters were continuously recorded throughout the experiment.

**Main results:**

Both treatment groups had similar time of ROSC, survival rates, hemodynamic and respiratory parameters during cardiopulmonary resuscitation. The hemodynamic recovery in the subsequent 4h was similar in both groups and was only slightly lower than sham-operated piglets at the end of experiment.

**Conclusion:**

Newborn piglets resuscitated by SI+CC 120 did not show a significant advantage in ROSC, survival, and hemodynamic recovery as compared to those piglets resuscitated by SI+CC 90.

## Introduction

Although chest compressions (CC) are infrequent events in newborn infants (with an incidence of 0.08% in near-term and term deliveries, and 10% in very preterm deliveries)[1,2], outcome studies of preterm infants requiring cardiopulmonary resuscitation (CPR) or CC have reported high rates of mortality and neurodevelopmental impairment in surviving children[1,2]. The poor prognosis associated with resuscitations requiring CC and/or medications in the delivery room raises questions as to whether improved CPR techniques that are specifically tailored to the newborn could improve outcomes[3–5].

Current resuscitation guidelines recommend 120 events per minute, which comprises of 90 CC and 30 inflations[6]. However, the most effective method of delivering CC remains controversial. Wyckoff and Berg suggested that the current 3:1 CPR does not achieve adequate coronary perfusion, and that further optimization of CC is required. Continuous CC has been shown to avoid interrupting coronary perfusion and may improve minute ventilation during CPR[7]. In comparison, animal studies and adult human randomized trials have demonstrated that continuous CC without rescue breaths increase return of spontaneous circulation (ROSC) and survival after sudden cardiac collapse[8–10]. The increase in survival and ROSC was predominately related to an increase in intrathoracic pressure generated by continuous CC[11]. A further technique to increase intrathoracic pressure is the delivery of a sustained inflation (SI)[12,13]. Studies have demonstrated that SI can i) achieve development of functional residual capacity during initial resuscitation of newborn infants more rapidly[14,15], and ii) increase carotid blood flow during CC[16–19]. We recently described a novel CPR technique delivering CC superimposed by SI during CPR in newborn piglets, which significantly improved ROSC and survival in newborn piglets[20]. The study used a CC rate of 120/min, which is different form the current neonatal resuscitation recommendation. In fact, a mathematical study suggested that the most effective CC frequency during CPR depends on body size and weight[21]. CC rates >120/min might be more beneficial for newborn infants and may improve survival[21]. However, the optimal rate of CC during SI has not yet been established. Our aim was to determine whether different CC rates during SI reduced time to ROSC in newborn piglets with asphyxia-induced bradycardia. We hypothesized that using a rate of 90/min would achieve a similar time to ROSC and survival compared to using 120/min.

## Methods

**Fig 1:**
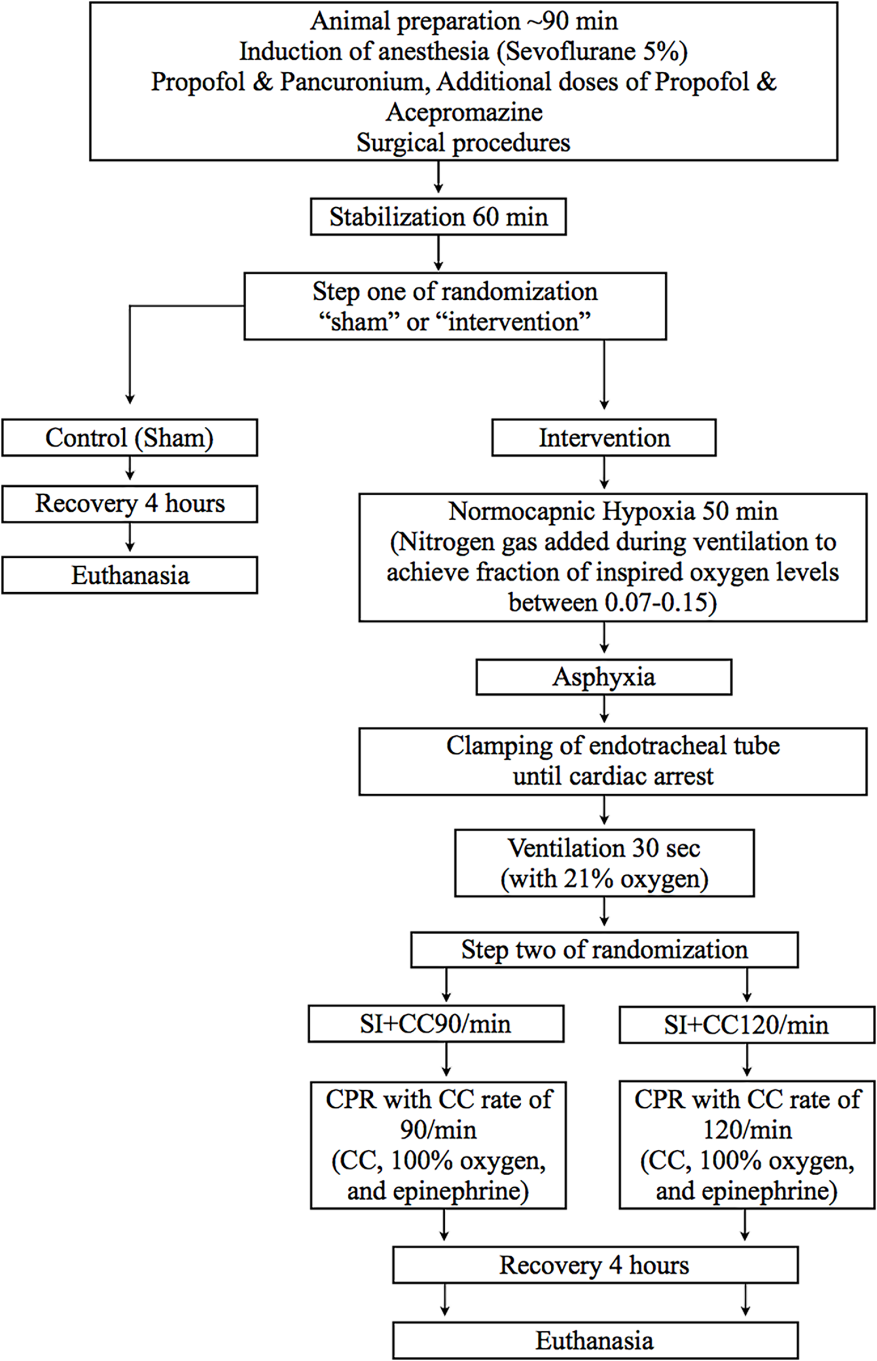
Study flow chart.

Twenty-two newborn mixed breed piglets (1–5 days of age, weighing 1.7–2.3 kg) were obtained on the day of experimentation from the University Swine Research Technology Centre. All experiments were conducted in accordance with the guidelines and approval of the Animal Care and Use Committee (Health Sciences), University of Alberta and presented according to the ARRIVE guidelines (S1 File) [22]. A graphical display of the protocol is presented in Fig 1 and the completed ARRIVE guidelines have been submitted in S1 File.

### Randomization

Piglets were randomly allocated to sham-operated, or SI groups. Allocation was block randomized with variable sized blocks (2 to 4) using a computer-generated randomization program (http://www.randomizer.org). Sequentially numbered, sealed, opaque envelopes containing the allocation were opened during the experiment (Fig 1).

### Animal preparation

Piglets were instrumented as previously described with modifications[20,23]. Following the induction of anaesthesia using isoflurane, piglets were intubated via a tracheostomy, and pressure-controlled ventilation (Sechrist infant ventilator, model IV-100; Sechrist Industries, Anaheim, CA) was commenced at a respiratory rate of 16–20 breaths/min and pressure of 20/5 cmH_2_O. Oxygen saturation was kept within 90–100%, glucose level and hydration was maintained with an intravenous infusion of 5% dextrose at 10 mL/kg/hr. During the experiment anaesthesia was maintained with intravenous propofol 5–10 mg/kg/hr and morphine 0.1 mg/kg/hr. Additional doses of propofol (1–2 mg/kg) and morphine (0.05–0.1 mg/kg) were also given as needed. The piglet’s body temperature was maintained at 38.5–39.5°C using an overhead warmer and a heating pad[24].

### Hemodynamic parameters

A 5-French Argyle^®^ (Klein-Baker Medical Inc. San Antonio, TX) double-lumen catheter was inserted via the right external jugular vein for administration of fluids and medications. A 5-French Argyle^®^ single-lumen catheter was inserted above the right renal artery via the femoral artery for continuous arterial blood pressure monitoring in addition to arterial blood gas measurements. A Millar catheter (MPVS Ultra^®^, ADInstruments, Houston, TX) was inserted into the left ventricle via the left common carotid artery for continuous measurement of left ventricular pressure, composite and segmental volumes, which were used for cardiac output calculation. The right common carotid artery was also exposed and encircled with a real-time ultrasonic flow probe (2mm; Transonic Systems Inc., Ithica, NY) to measure blood flow.

Piglets were placed in supine position and allowed to recover from surgical instrumentation until baseline hemodynamic measures were stable. Ventilator rate was adjusted to keep the partial arterial CO_2_ between 35–45 mmHg as determined by periodic arterial blood gas analysis. Mean systemic arterial pressure, systemic systolic arterial pressure, heart rate, and percutaneous oxygen saturation were continuously measured and recorded throughout the experiment with a Hewlett Packard 78833B monitor (Hewlett Packard Co., Palo Alto, CA).

### Respiratory parameters

A respiratory function monitor (NM3, Respironics, Philips, Andover, MA) was used to continuously measure tidal volume (V_T_), airway pressures, gas flow, end-tidal CO_2_ (ETCO_2_). The combined gas flow and ETCO_2_ sensor was placed between the endotracheal tube and the ventilation device. V_T_ was calculated by integrating the flow signal. ETCO_2_ was measured using non-dispersive infrared absorption technique. The accuracy for gas flow is ±0.125 L/min, ETCO_2_ ±2 mmHg[25].

### Cerebral perfusion

Cerebral oxygenation (crSO_2_) was measured using the Invos^™^ Cerebral/Somatic Oximeter Monitor (Invos 5100, Somanetics Corp., Troy, MI). The sensors were placed on the right forehead of the piglet and secured with wrap and tape. Light shielding was achieved with a slim cap. The Invos^™^ Cerebral/Somatic Oximeter Monitor calculates crSO_2_, which is expressed as the percentage of oxygenated haemoglobin (oxygenated haemoglobin/total haemoglobin). Values of regional oxygen saturation are stored every second with a sample rate of 0.13 Hz[26].

### Experimental protocol

Piglets were randomized into three groups: CC superimposed by SI at a rate of 90 CC per minute (SI+CC 90, n = 8), CC superimposed by SI at a rate of 120 CC per minute (SI+CC 120, n = 8), or a sham group (n = 6). Two-step randomization was used to reduce selection bias. After surgical instrumentation and stabilization, a sequentially numbered, sealed brown envelope containing the allocation “sham” or “intervention” was opened (step one) (Fig 1). Only piglets randomized to “intervention” underwent hypoxia and asphyxia. The sham-operated group had the same surgical protocol, stabilization, and equivalent experimental periods without hypoxia and asphyxia. Once the criteria for CPR were met, a second sequentially numbered, sealed brown envelope containing the allocations “SI+CC 90” or “SI+CC 120”, was opened (step two) (Fig 1). Both treatment groups received SI with a peak inflating pressure of 30 cm H_2_O for a duration of 30 sec. SI were then interrupted for a one second expiratory pause before a further 30 sec of SI were provided. During the SI, CC was performed at a rate of 90/min or 120/min. Ventilation rate used in the two models of SI+CC were 90/min in the SI+CC 90 and 120/min the SI+CC 120, respectively (ventilation rate = chest compression rate).

Piglets randomized to “intervention” were exposed to 50 minutes of normocapnic hypoxia. Hypoxia was followed by asphyxia until heart rate decreased to 25% of baseline, which was achieved by disconnecting the ventilator and clamping the endotracheal tube. Ten seconds after heart rate reached 25% of baseline, positive pressure ventilation (PPV) was commenced for 30 seconds with a Neopuff T-Piece (Fisher & Paykel, Auckland, New Zealand). The default settings were a peak inflating pressure of 30 cmH_2_O, a positive end expiratory pressure of 5 cmH_2_O, and a gas flow of 8 L/min. CC were performed using the two-thumb encircling technique by a single operator (LM) in all piglets. A metronome was used to achieve the targeted CC rate. After 30 seconds of CC, 100% oxygen was commenced. Epinephrine (0.01 mg/kg per dose) was administered intravenously 1 minute after the start of PPV, and administered every minute as needed if no increase in heart rate or ROSC was observed despite adequate ventilation and CC. Epinephrine was administered to a maximum of 4 doses. ROSC was defined as an unassisted heart rate ≥100 bpm for 15 seconds. After ROSC, piglets were allowed to recover for four hours before the piglets were euthanized with an intravenous overdose of phenobarbital (100 mg/kg).

### Sample size and power estimates

Our primary outcome measure was CPR time to achieve ROSC. Our previous studies showed a mean ±standard deviation ROSC of 180±25 seconds during CPR using 3:1 C:V. Given Babbs et al[21] postulated that higher CC rates would improve outcomes, we hypothesized that the use of SI during CPR with SI+CC120/min would reduce time to achieve ROSC. A sample size of 16 piglets (8 per group) was sufficient to detect a clinically important (33%) reduction in time to achieve ROSC (i.e. 180 sec vs. 120 sec), with 80% power and a 2-tailed alpha error of 0.05.

### Data collection and analysis

Demographics of study piglets were recorded. Transonic flow probes, heart rate and pressure transducer outputs were digitized and recorded with custom Asyst programming software (Data Translation, Ontario, Canada). Airway pressures, gas flow, V_T_, and ETCO_2_ were measured and analysed using Flow Tool Physiologic Waveform Viewer (Philips Healthcare, Wallingford, CT, USA). The data are presented as mean ±standard deviation (SD) for normally distributed continuous variables and median (interquartile range—IQR) when the distribution was skewed. For all respiratory parameters, continuous values during CPR were analysed. The data was tested for normality and compared using Student’s *t-test* for parametric and Mann-Whitney *U*-test for nonparametric comparisons of continuous variables, and χ^2^ for categorical variables. *P*-values are 2-sided and p<0.05 was considered statistically significant. Statistical analyses were performed with SigmaPlot (Systat Software Inc, San Jose, USA).

## Results

Twenty-two newborn mixed breed piglets were obtained on the day of the experiment, and were randomly assigned to the SI+CC 90 group, the SI+CC 120 group, and the sham-operated group. There were no differences in the baseline parameters between the groups (Table 1).

**Table 1:** Baseline characteristics.

	Sham (n = 6)	SI + CC90 (n = 8)	SI + CC120 (n = 8)	p-value
Age (days)	2.0 (1.0–2.0)	2.0 (1.5–3.5)	2.0 (1.5–2.5)	0.62
Weight (kg)	2.0 (1.7–2.2)	2.0 (1.9–2.2)	2.0 (1.9–2.0)	0.84
Heart rate (bpm)	202 (187–222)	238 (223–249)	221 (205–246)	0.07
MAP (mmHg)	81 (69–89)	74 (64–91)	79 (72–89)	0.81
CO (mL/kg/min)	231 (198–306)	334 (308–378)	201 (172–251)	0.18
Ejection Fraction (%)	27 (27–30)	27 (23–40)	21 (14–27)	0.08
Max dp/dt (mmHg)	3984 (3308–6031)	6719 (5187–8045)	5265 (3665–6325)	0.09
Tau (ms)	19 (18–21)	14 (13–16)	15 (13–19)	0.48
Carotid flow (mL/min/kg)	30 (27–33)	41 (33–47)	37 (32–43)	0.16
Cerebral oxygenation (%)	38 (34–40)	44 (43–51)	43 (39–46)	0.15
pH	7.4 (7.4–7.4)	7.4 (7.3–7.4)	7.4 (7.3–7.4)	0.73
Base excess (mmol/L)	-2 (-2~1)	-4 (-7~-2)	-1(-6~2)	0.32
paCO_2_ (torr)	41 (39–46)	39 (35–43)	41 (36–42)	0.89
SpO_2_ (%)	91 (90–92)	92 (88–93)	92 (90–93)	0.82
Lactate (mmol/L)	3.2 (3.0–4.23)	3.8 (3.5–4.6)	3.1 (2.5–4.4)	0.47
Arterial hemoglobin (g/L)	78 (64–84)	76 (71–82)	77 (70–84)	0.82

Data are presented as median (IQR); MAP- Mean arterial blood pressure, CO—Cardiac output

### Resuscitation

As shown in Table 2, there was no difference in asphyxia time as well as degree of asphyxiation (as indicated by pH, PaCO_2_ and lactate) between the two asphyxiated piglet groups. During CPR, 3/8 piglets in the SI+CC 90 group and 5/8 piglets in the SI+CC 120 group required 100% oxygen, and 3/8 piglets in the SI+CC 90 group and 6/8 piglets in the SI+CC 120 group received epinephrine (p = 0.32). Consequently, there was no difference in time to ROSC between the SI+CC 90 group and the SI+CC 120 group (Table 2). Survival rate was 7/8 piglets vs. 6/8 piglets in the SI+CC 90 and SI+CC 120 group respectively (Table 2). Three piglets (1 in the SI+CC 90 and 2 in the SI+CC 120 group) did not achieve ROSC and died (they were euthanized with intravenous overdose of phenobarbital (100 mg/kg)).

**Table 2:** Characteristics of asphyxia, resuscitation and survival of asphyxiated piglets (n = 8 in each group)

	SI + CC90	SI + CC120	p-value
Asphyxia time (sec)	68 (50–158)	98 (53–165)	0.66
Immediately before resuscitation			
pH	6.9 (6.8–6.9)	6.9 (6.8–7.0)	0.80
paCO_2_ (torr)	64 (54–72)	64 (56–87)	0.37
Lactate (mmol/L)	13.8 (12.8–14.4)	13.9 (12.4–14.4)	0.74
Resuscitation			
Received 100% oxygen (n(%))	3 (38)	5 (63)	0.62
Epinephrine doses (n)	0 (0–2)	1 (0–4)	0.41
Achieving ROSC (n (%))	7 (88)	6 (75)	0.98
ROSC time (sec)	34 (28–156)	99 (31–255)	0.29
Survival 4h after ROSC (n (%))	7 (88)	6 (75)	0.98

Data are presented as median (IQR); ROSC—return of spontaneous circulation, “Immediately before resuscitation” refers to the time of cardiac arrest immediately before positive pressure ventilation was initiated

### Respiratory Parameters during CC

During chest compression there were no difference in delivered V_T_, gas flow or peak inflation pressure between groups (Table 3). As expected there was a significant difference in the numbers of delivered inflations (which equals chest compression) between the groups (Table 3). Although, minute ventilation was similar between groups, the SI+CC 90 had a higher V_T_. The lower V_T_ delivered in the SI+CC 120 group might be due to higher dead space and/or shorter inflation time (with the higher ventilation rate). There was no difference between the fraction of inspired oxygen (p = 0.63) or the partial pressure of arterial oxygen saturation (p = 0.295) between the two intervention groups.

**Table 3:** Respiratory parameters before ROSC.

	SI+CC 90/min	SI+CC 120/min	p-value
Chest compression rate (/min)	90 (1)	115 (9)	<0.001
Ventilation rate (/min)	90 (1)	115 (9)	<0.001
Tidal volume (mL/kg)^#^	15 (10.7–16.7)	12 (10.9–13.8)	0.25
Minute ventilation in mL/kg/min	632 (196)	681 (133)	0.55
Peak inflation pressure (cm H_2_O)	33.7 (1.2)	33.3 (0.9)	0.44
Peak inspiratory flow	7.8 (1.7)	7.9 (0.9)	0.90
Peak expiratory flow	14 (2.9)	13.7 (1.9)	0.76
End-tidal CO_2_ (mm Hg)	28 (17–38)	20 (11–30)	0.29

Data are presented as mean (SD) unless indicated ^#^median (IQR); V_T−_tidal volume, MV—minute ventilation, ECO_2_ –exhaled CO_2_

### Changes in Hemodynamic Parameters

**Fig 2:**
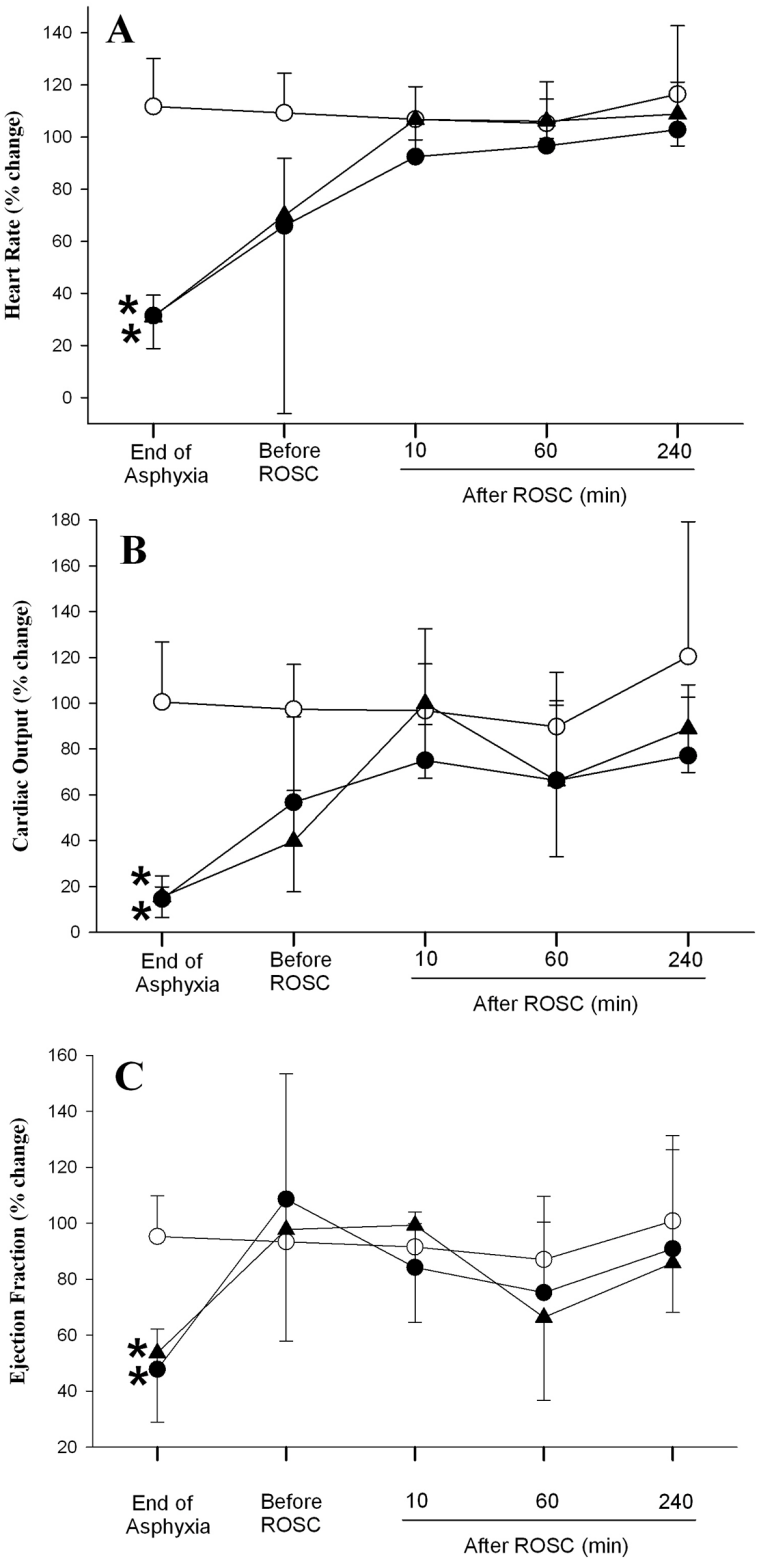
Percent changes from normoxic baseline in (A) heart rate, (B) cardiac output, and (C) ejection fraction in sham (◯), SI+CC 90 (●), and SI+CC 120 (▴) groups during and after resuscitation. Each point represents mean±SD. *Significantly different from sham-operated group, p<0.05 (Tukey).

**Fig 3:**
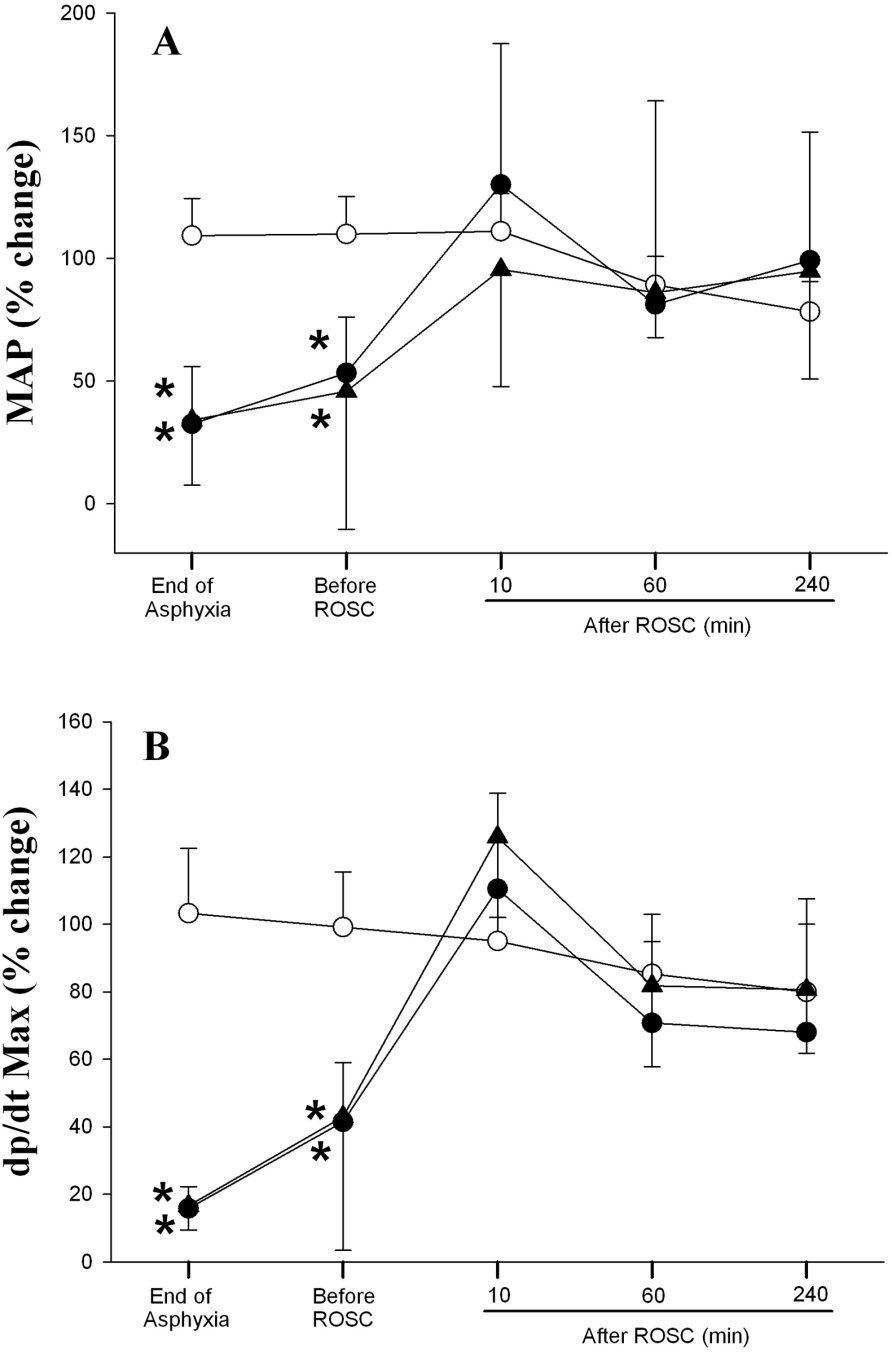
Percent changes from normoxic baseline in (A) mean arterial pressure (MAP), and (B) dp/dt max in sham (◯), SI+CC 90 (●), and SI+CC 120 (▴) groups during and after resuscitation. Each point represents mean±SD. *Significantly different from sham-operated group, p<0.05 (Tukey).

**Fig 4:**
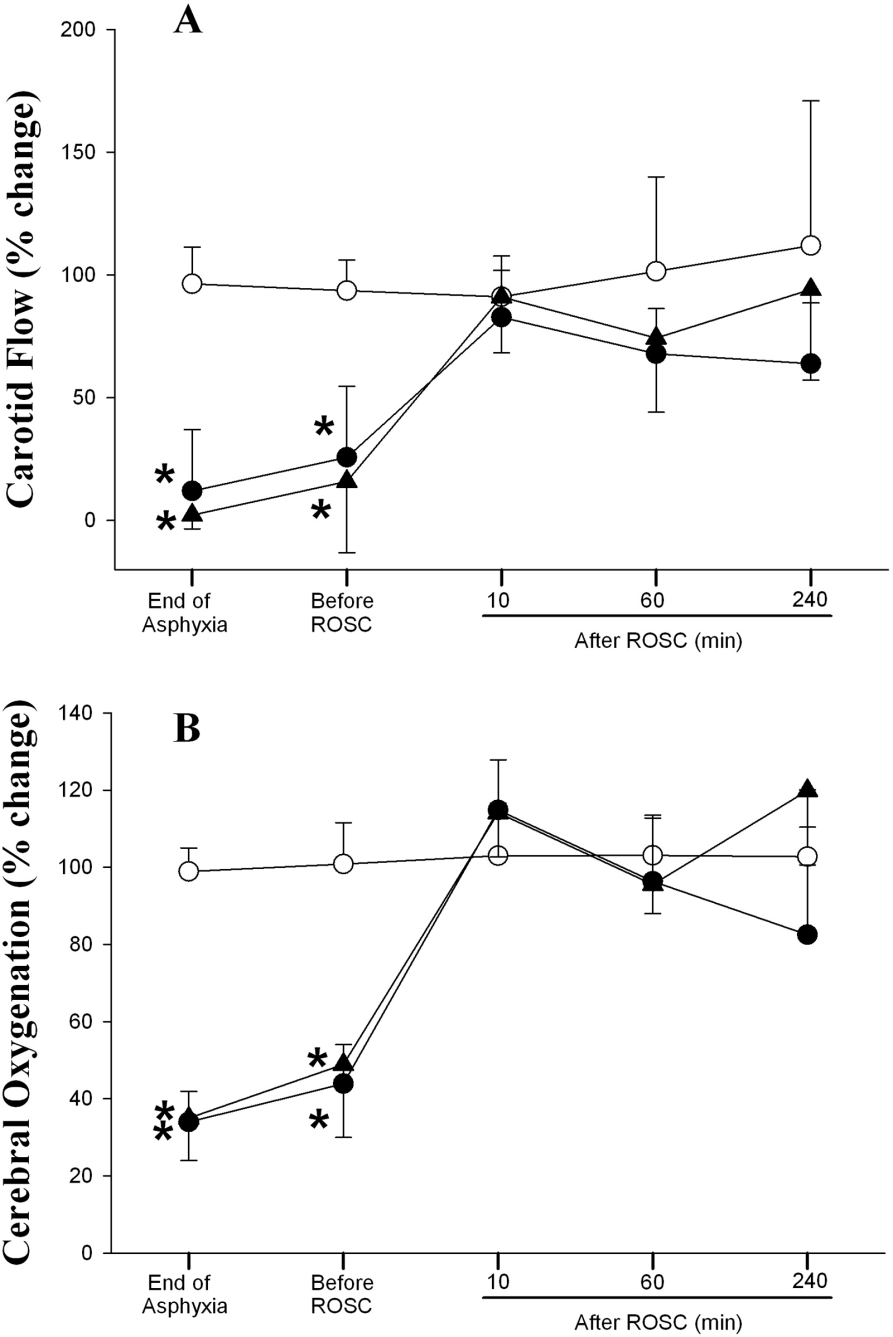
Percent changes from normoxic baseline in (A) carotid flow, and (B) cerebral oxygenation in sham (◯), SI+CC 90 (●), and SI+CC 120 (▴) groups during and after resuscitation. Each point represents mean±SD. *Significantly different from sham-operated group, p<0.05 (Tukey).

Hemodynamic changes of all experimental groups are summarized in Figs 2–4. Generally, there was no difference in all hemodynamic parameters examined between the two asphyxia/resuscitation groups.

Fig 2 shows changes in heart rate, cardiac output and ejection fraction during asphyxia, 10 sec before ROSC and after resuscitation. Heart rate of both experimental groups decreased to 25% of baseline value by the end of asphyxiation and it increased drastically to around 70% of baseline value immediately before ROSC. Heart rate was maintained around the baseline value throughout the reminder of the experimental period. By the end of asphyxiation, the cardiac output of both experimental groups was below 20% of baseline value (Fig 2B). It increased to around 55 and 40% of baseline value for SI+CC 90 and SI+CC 120 groups, respectively. Even though the cardiac output of both groups was lower than that of the sham control group after resuscitation, the difference did not achieve any statistical significance (Fig 2B). As shown in Fig 2C, the ejection fraction of both experimental groups was significantly lower than that of the sham control group by the end of asphyxia. It increased back to the baseline value before ROSC and was similar to sham control values after resuscitation.

Changes in MAP and dp/dt max are summarized in Fig 3. The MAP of both treatment groups decreased significantly at the end of asphyxia and shortly before ROSC. It then increased to approximately the baseline value immediately after resuscitation and remained similar to sham controls throughout the experimental period (Fig 3A). The pattern of dp/dt max of both experimental groups was similar to that observed with MAP changes (Fig 3B).

As shown in Fig 4A, the carotid flow of both treatment groups was significantly lower than the sham control group at the end of asphyxia and shortly before ROSC. Immediately following resuscitation, the carotid flow returned to the baseline value where it was maintained for the remainder of the experimental period. Changes in cerebral reoxygenation in both experimental groups followed a similar pattern to that of the carotid flow (Fig 4B).

## Discussion

A mathematical study suggested that the most effective CC frequency during CPR depends on body size and weight, and that CC rates >120/min might be more beneficial for newborn infants and may improve survival[21]. In a recent study by our group we compared the standard 3:1 technique using 90 CC/minute and compared it with CC superimposed by SI using 120 CC/minute[20]. The higher CC rate, as postulated by Babbs et al[21], could have contributed to the faster ROSC time in the SI+CC group. However, the real effect of different CC rates when superimposed by SI remains unknown. In the current study we compared CC rates of 90/min vs. 120/min during SI in a porcine model of neonatal asphyxia. The results of the study can be summarized as: 1) during SI+CC 90 ROSC time was 34 sec compared to 99 sec in the SI+CC 120 group, although not statistically significant ROSC was only a third in the SI+CC90 group (Table 2); 2) overall survival to achieve ROSC and in the following 4 hours after ROSC were similar between the groups (Table 2); 3) tidal volume delivery, minute ventilation, hemodynamic recovery and cerebral oxygenation were similar between the groups. Our study is the first to examine if an increased CC rate can improve survival. However, increasing CC rate during SI did not provide any advantage in overall resuscitation outcomes in our newborn piglet model. Similar studies using either 9:3 or 15:2 C:V ratio in asphyxia-induced cardiac arrest piglets also did not report an improvement in ROSC[27,28]. Similarly, no difference in various hemodynamic parameters in infant piglets receiving either continuous chest compression or 5:1 C:V ratio has been reported^29^. These studies suggest that a rate of 90/min might be sufficient during neonatal CPR. Furthermore, despite a similar systemic hemodynamic recovery, the SI+CC 120 group had modestly higher cerebral oxygenation (vs. SI+CC 90 group, p<0.05), which could be related to the higher carotid blood flow. However, we have no information regarding neuronal damage including the levels of S-100 and NSE.

It remains controversial how the blood flows during chest compression. Berkowitz et al postulated that conventional CPR in small animals generated sufficient intrathoracic pressure to achieve forward blood flow because of the shape and compliance of their chest[29]. However, several studies demonstrated that an adequate increase in forward blood flow is achieved when several techniques to increase intrathoracic pressure (e.g. CC and High ventilation pressures) are combined[16,30]. We have recently demonstrated that significant improvements in ROSC and hemodynamic recovery can be observed in piglets when given continuous CC superimposed with SI compared to 3:1 C:V CPR[20]. The beneficial effect of combining two techniques that increase intrathoracic pressure resulted in improved respiratory and hemodynamic parameters[20]. A detailed analysis of the respiratory parameters also revealed an improved tidal volume delivery during CC+SI compared to 3:1 C:V, resulting in better tissue oxygenation during resuscitation[31]. In the current study the tidal volume delivery was slightly smaller in the SI+CC 120 group (not statistically significant–Table 3), which resulted in a similar minute ventilation, despite a significantly higher CC rate. This might be due to higher dead space or shorter inflation time (with the higher ventilation rate) resulting in lower V_T_ and lower ETCO_2_ (Table 3). In addition, the ETCO_2_ was slightly lower in the SI+CC 120 group (again not statistically significant–Table 3), which suggest that higher CC rates might impair gas exchange and rates ~90/min are sufficient to deliver an adequate V_T_ and minute ventilation without impairing gas exchange. However, further studies on the optimal inflation pressure to maintain an effective functional residual capacity as well as achieve adequate V_T_ delivery during CC+SI are warranted.

## Limitations

Our use of a piglet asphyxia model is a great strength of this translational study, as this model closely simulates delivery room events, with the gradual onset of severe asphyxia leading to bradycardia[32,33]. However, several limitations should be considered before implementing simultaneous CC and SI in the delivery room. Our asphyxia model uses piglets that have already undergone the fetal to neonatal transition, and piglets were sedated/anesthetized. Furthermore, our model requires piglets to be intubated with a tightly sealed endotracheal tube to prevent any endotracheal tube leak; this may not occur in the delivery room as mask ventilation is frequently used. Nevertheless, our findings are still clinically relevant as the distribution of cardiac output in the fetus and posttransitional neonate during asphyxia episodes are qualitatively similar[34]. Our resuscitation model is slightly different from the currently recommended resuscitation guidelines, as we gave 100% oxygen after 30sec of CC, and administered epinephrine 60sec after CC were initiated, and gave an additional dose every minute[6]. This may have influenced our results. Nevertheless, there was no significant difference in the amount of epinephrine doses between both groups. Although, the time of ROSC was faster in the SI+CC 90 group, unfortunately this was not statistically significant. However, we believe a 2/3 reduction in duration of CC would be clinically significant. However the lack of statistical significance could be due to the small sample size.

## Conclusion

Using a higher CC rate during SI did not provide any advantage on ROSC, survival rate or hemodynamic recovery during neonatal resuscitation in a porcine mode of asphyxia.

## Supporting Information

S1 File (DOC)
